# Association between time-related work factors and dietary behaviors: results from the Japan Environment and Children’s Study (JECS)

**DOI:** 10.1186/s12199-018-0753-9

**Published:** 2018-12-14

**Authors:** Rie Tanaka, Mayumi Tsuji, Koichi Kusuhara, Toshihiro Kawamoto, Japan Environment and Children’s Study Group, Toshihiro Kawamoto, Hirohisa Saito, Reiko Kishi, Nobuo Yaegashi, Koichi Hashimoto, Chisato Mori, Shuichi Ito, Zentaro Yamagata, Hidekuni Inadera, Michihiro Kamijima, Takeo Nakayama, Hiroyasu Iso, Masayuki Shima, Yasuaki Hirooka, Narufumi Suganuma, Koichi Kusuhara, Takahiko Katoh

**Affiliations:** 1 Department of Environmental Health, School of Medicine, University of Occupational and Environmental Health, 1-1 Iseigaoka, Yahatanishi-ku, Kitakyushu, Fukuoka 807-8555 Japan; 2 Japan Environment and Children’s Study, UOEH Subunit Center, University of Occupational and Environmental Health, 1-1 Iseigaoka, Yahatanishi-ku, Kitakyushu, Fukuoka 807-8555 Japan; 3 Department of Pediatrics, School of Medicine, University of Occupational and Environmental Health, 1-1 Iseigaoka, Yahatanishi-ku, Kitakyushu, Fukuoka 807-8555 Japan

**Keywords:** Workhours, Shift work, Occupation, Dietary behaviors

## Abstract

**Background:**

Few studies have examined the association of workhours and shift work (referred to here as “time-related work factors”) with dietary behaviors. We aimed to investigate this association, as well as the dietary behaviors among individuals with occupations characterized by time-related work factors.

**Methods:**

A cross-sectional study was performed using data from the Japan Environment and Children’s Study. The study included 39,315 working men. Dietary behaviors (i.e., skipping breakfast, eating out, eating instant food, overeating, and eating fast) were assessed with a self-reported information from the Food Frequency Questionnaire. Logistic regression analysis was conducted to examine the associations of time-related work factors with dietary behaviors and dietary behavior tendencies among those in occupations characterized by long workhours and/or shift work.

**Results:**

Long workhours were associated with high frequencies of skipping breakfast, eating out, eating instant food, overeating, and eating fast. The frequency of having shift work was associated with high frequencies of skipping breakfast, eating out, and eating instant food. Several occupations involving long workhours and/or shift work showed specific dietary behaviors; in some occupations, the level of significance changed after adjusting for time-related work factors in addition to other potential confounding factors.

**Conclusions:**

Time-related work factors may help explain workers’ dietary behaviors. Long workhours and shift work may lead to poor dietary behaviors. Other factors influenced by occupation itself, such as food environment, may also influence workers’ dietary behaviors. Workhours and/or shift work, and these other work factors, should be given attention in workplace health promotion.

## Background

The association between working conditions and health has been widely recognized. Particularly, time-related work factors, such as workhours and shift work, have been reported to be associated with health issues, including obesity [1], metabolic syndrome [2], and cardiovascular disease [3]. It is crucial to develop an appropriate approach for shift workers and those working long hours to prevent diseases and promote health.

The association between workhours/shift work and health may be partially attributed to workers’ dietary behaviors. According to a previous study conducted in various EU countries, irregular workhours was the most frequently reported barrier for healthy eating [4]. Another study including young adults showed that men working more than 40 h per week were more likely to report time-related barriers to healthy eating, such as “too rushed in the morning to eat a healthy breakfast” and “eating healthy meals takes too much time” [5]. The association between shift work and irregular eating patterns has also been well documented [6].

Dietary behaviors associated with time-related work factors can vary across occupations; thus, knowledge of differences in dietary behaviors between occupations is necessary to make progress in workplace health promotion. Few studies, however, have examined the differences in dietary behaviors between occupations. Relatively poor dietary behaviors have been observed in specific occupations, including among health professionals (physicians [7], nurses [8]), service workers [9], transportation workers [9], and laborers [9]. The exact cause of unhealthy dietary behaviors remains unknown.

Therefore, we hypothesized that the presence of poor dietary behaviors among specific occupations could be attributed to time-related work factors (referred to here as “workhours” and “shift work”). The present study focused on male workers; men’s diets may be more likely to be affected by time-related work factors than women’s diets. According to a previous study in Europe, men reported “irregular workhours” more frequently as a barrier to healthy eating than women [4]. Gender differences in diet, such as nutrition knowledge [10], attitude [10], behavior [10], and dietary pattern [11], have also been reported previously; for example, one study in Japan reported that compared to women, men showed some dietary patterns, such as lower score for “high-bread and low-rice” and “vegetable” and higher score for “high-meat and low-fish” [11].

The aims of this study were as follows: to provide an overview of workers’ dietary behaviors according to their workhours and presence of shift work and to examine their dietary behaviors according to the occupations involving working long hours and/or shift work.

## Methods

### Study design

This study was based on baseline data from the Japan Environment and Children’s Study (JECS) (jecs-ag-20160424), which was released in June 2016 [12]. The JECS was designed to investigate the influence of environmental factors on children’s health. From the 15 regional centers located across Japan, more than 100,000 pregnant women (mothers) were recruited to the JECS from January 2011 to March 2014, with optional participation being extended to their partners (fathers). The JECS was approved by the Institutional Review Board of the Japan National Institute for Environmental Studies (Approval number: 2017-002) and the Ethics Committees of all participating institutions. The study was conducted in accordance with the Declaration of Helsinki and other national regulations. Written informed consent was obtained from all study participants. Details on the study protocol have been reported previously [13, 14].

### Study participants

**Fig. 1:**
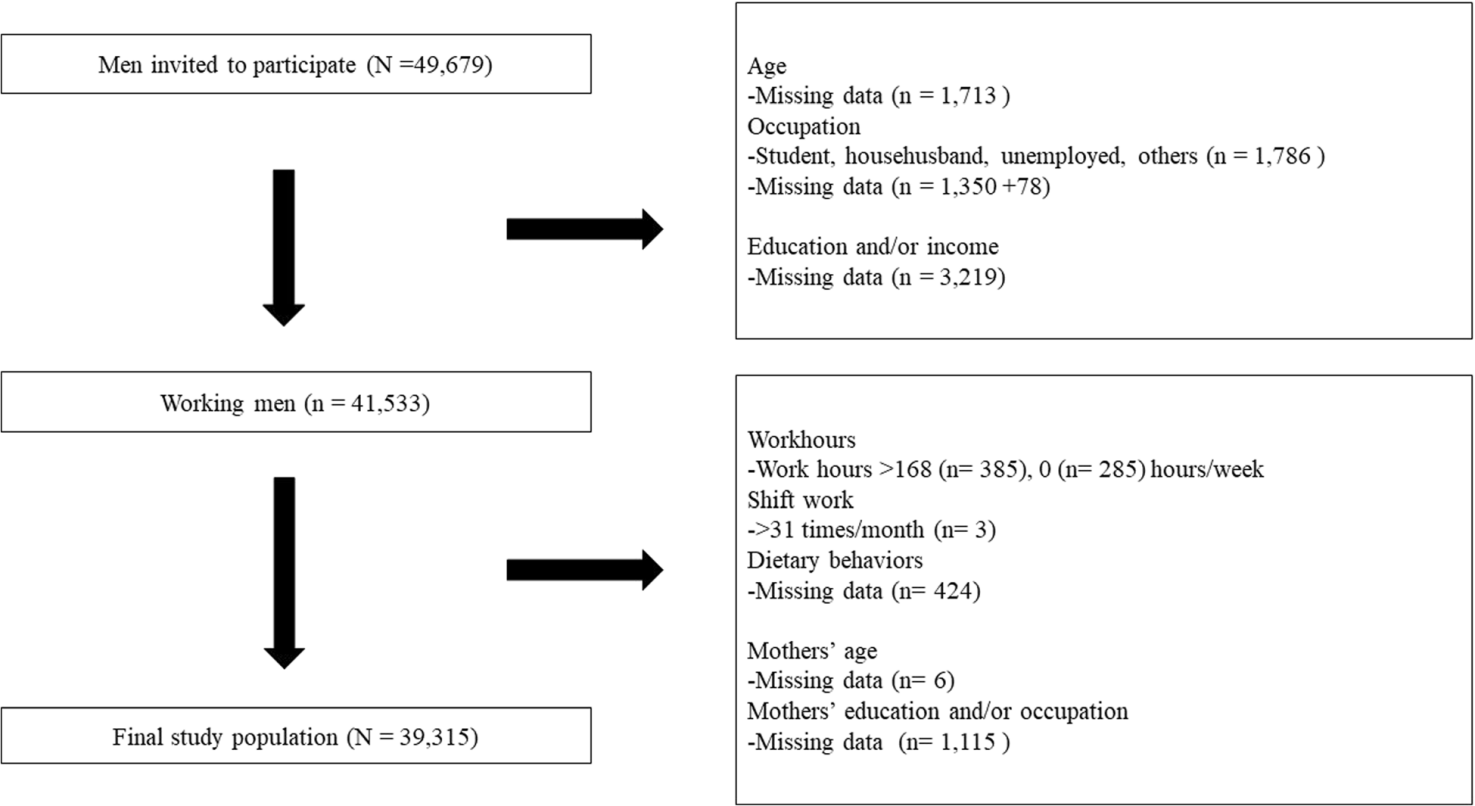
Participant inclusion flowchart

Information on workhours, shift work, and occupations were obtained from self-administered questionnaires provided to male participants (fathers). Information on partners’ occupation (mothers’ occupation) was obtained from self-administered questionnaires provided to female participants (mothers) during their first trimester. Information about household income, educational level (fathers’ education), and partners’ educational level (mothers’ education) were obtained from self-administered questionnaires provided to female participants (mothers) during the second or third trimester of their pregnancy. Participants (fathers) who reported their occupations as “students,” “househusbands,” “unemployed,” or “workers not otherwise classifiable” and those with missing questionnaire data were excluded. Finally, 39,315 men were included in the analysis. The flowchart of the selection process is shown in Fig. 1.

### Dietary behaviors

Participants’ dietary intakes were assessed using the Food Frequency Questionnaire (FFQ) that was used in the Japan Public Health Center-Based Prospective Study for the Next Generation and has been validated previously [14, 15]. The following dietary behaviors were assessed using five questions from the FFQ: “How often do you eat breakfast?”; “How often do you eat out?”; “How often do you eat instant food?”; “Do you tend to overeat?”; and “Do you tend to eat fast?”

Response options regarding eating breakfast, eating out, and eating instant food were as follows: “less than once a month,” “one to three times per month,” “one to two times per week,” “three to four times per week,” “five to six times per week,” and “every day.” The response options regarding eating out and eating instant food were categorized into “less than once a month,” “one to three times per month”, and “one to two times per week” vs “three to four times per week,” “five to six times per week,” and “every day.” The response options regarding eating breakfast were categorized into “every day” vs “less than once a month,” “one to three times per month,” “one to two times per week,” “three to four times per week,” and “five to six times per week” (hereinafter called “skipping breakfast”). Response options regarding overeating and eating fast were “no” vs “yes.”

### Time-related work factors: workhours and shift work

The number of weekly workhours was calculated from the answers to the questions “How many hours do you work per day?” and “How many days do you work per week?” Accordingly, workhours were categorized into six groups: equal to or less than 40 h; > 40, ≦ 45 h; > 45, ≦ 50 h; > 50, ≦ 55 h; > 55, ≦ 65 h; and more than 65 h per week. Information on shift work was assessed using the question: “How often do you have shifts other than the day shift?” Based on the responses, the frequency of shift work was categorized into three groups: no (with “zero” as the answer), > 0, ≦ 8 times, and more than 8 times per month.

### Socioeconomic factors

Participants’ educational levels and mothers’ educational levels were categorized into junior high school, high school, higher professional school, professional school, junior college, university, and graduate school. Their annual income was categorized into < 2 million yen, 2.0–3.9 million yen, 4.0–5.9 million yen, 6.0–7.9 million yen, 8.0–9.9 million yen, 10.0–11.9 million yen, 12.0–14.9 million yen, 15.0–19.9 million yen, and ≥ 20 million yen. Participants’ occupation was classified using the Japanese Occupational Classification (Rev. 5, December 2009) [16], which contains 12 major groups of workers: administrative and managerial; professional and engineering; clerical; sales; service; security; agricultural, forestry, and fishery; manufacturing; transport and machine operation; construction and mining; carrying, cleaning, packaging, and related work; and workers not classifiable by occupation (who were not included in the present analysis). Furthermore, occupation was classified into smaller and more specific groups using the more detailed classification based on the Minor Groups or Unit Groups of the Japanese Standard Occupational Classification (Rev. 5, December 2009) [16, 17]. This study used the latter classification. Small groups comprising less than 1.0% of all participants were integrated into other groups or unified within each major group. For example, within the “administrative and managerial workers” group, smaller groups comprising less than 1.0% of all participants were classified as “other administrative and managerial workers.” Within the “professional and engineering workers” group, smaller groups comprising less than 1.0% of all participants were classified as “other specialist professionals.” Within the “transport and machine operation workers” group, smaller groups consisting of less than 1.0% of all participants including stationary and construction machinery operators were integrated into a category for “other transport workers.” Forestry and fishery workers were unified in the same group. Carrying, cleaning, and packaging workers were also unified in the same group. Finally, the occupations were categorized into 39 groups. Partners’ occupation (mothers’ occupation) was classified into two groups: “12 major groups of workers (above mentioned)” vs “full-time homemaker,” “student and graduate student,” “unemployed,” and “workers not otherwise classifiable.”

### Statistical analysis

Logistic regression analysis was performed to examine the association between working conditions (workhours, shift work, and occupation) and dietary behaviors (skipping breakfast, eating out, eating instant food, overeating, and eating fast), using “less than 40 h per week” for workhours, “no” for shift work, and “management government officials” for occupation, as the reference categories, respectively. All the final models were adjusted for age, household income, educational level (fathers’ educational level), partners’ age (mothers’ age), partners’ educational level (mothers’ educational level), partners’ occupation (mothers’ occupation), and working conditions (workhours and/or shift work and/or occupations). All analyses were conducted using Stata/IC 14.0.

## Results

### Workhours and shift work by occupation

The characteristics of the study participants are shown in Table 1. Data on the workhours and the presence of shift work, by occupation, are shown in Table 2. The mean workhours per week were longer among doctors (68.0 ± 17.9 h/week), teachers (59.2 ± 14.4 h/week), food and drink preparatory workers (62.4 ± 15.4 h/week), judicial police staff such as police officers (59.7 ± 16.4 h/week), and motor vehicle drivers (63.5 ± 17.2 h/week). Shift work was more commonly observed among doctors (68.2%), nurses (85.7%), care service workers (65.5%), judicial police staff such as police officers (86.1%), and other public security workers, such as firefighters (78.5%). The mean frequency of shift work was much higher among merchandise sales workers (12.9 ± 8.0 times/month), food and drink preparatory workers (15.0 ± 9.0 times/month), customer service workers (13.9 ± 6.7 times/month), forestry workers and fishery workers (16.6 ± 8.1times/month), and motor vehicle drivers (13.9 ± 7.4 times/month).

**Table 1:** Characteristics of the study participants (*N* = 39,315)

	Mean	SD
Age (fathers’ age)	32.9	5.7
Educational level	*n*	%
Junior high school	1969	5.0
High school	14,018	35.7
Higher professional school	822	2.1
Professional school	7629	19.4
Junior college	846	2.2
University	11,993	30.5
Graduate school	2038	5.2
Household income		
< 2 million yen	1657	4.2
2.0–3.9 million yen	13,223	33.6
4.0–5.9 million yen	13,416	34.1
6.0–7.9 million yen	6572	16.7
8.0–9.9 million yen	2746	7.0
10–11.9 million yen,	1001	2.6
12–14.9 million yen	382	1.0
15–19.9 million yen	216	0.6
≥ 20 million yen	102	0.3
Breakfast eating		
Everyday	20,770	52.8
Not everyday	18,545	47.2
Eating outside		
≦ 3 times/month	16,789	42.7
≧ once/week	22,526	57.3
Instant food eating		
≦ 3 times/month	20,555	52.3
≧ once /week	18,760	47.7
Eating over		
No	11,341	28.9
Yes	27,974	71.2
Eating fast		
Normal to too slow	12,859	32.7
Too fast to slightly fast	26,456	67.3
Partners’ age (mothers’ age)	31.1	4.9
	*n*	%
Partners’ educational level (mothers’ education)		
Junior high school	1411	3.6
High school	11,557	29.4
Higher professional school	644	1.6
Professional school	9207	23.4
Junior college	7080	18.0
University	8768	22.3
Graduate school	648	1.7
Partners’ occupation (mothers’ occupation)		
Administrative and managerial workers	224	0.6
Professional and engineering workers	9637	24.5
Clerical workers	6889	17.5
Sales workers	2139	5.4
Service workers	5658	14.4
Security workers	116	0.3
Agricultural, forestry, and fishery workers	160	0.4
Manufacturing process workers	1356	3.5
Transport and machine operation workers	76	0.2
Construction and mining workers	30	0.1
Carrying, cleaning packaging, and related workers	163	0.4
Full-time homemaker	10,922	27.8
Student, graduate student	155	0.4
Unemployed	1214	3.1
Workers not otherwise classifiable	576	1.5

*SD* standard deviation

**Table 2:** Workhours and shift work according to occupations (*N* = 39,315)

		Workhours		Shift work			
	*n*	Mean	SD	Shift workers		Mean	SD
				*n*	%		
Total subjects	39,315	52.8	12.8	8867	22.6	9.7164	6.0
Occupations							
Administrative and managerial workers							
Management government officials	590	48.3	10.4	60	10.2	7	5.6
Officers of companies and organizations	396	58.5	16.4	37	9.3	10	8.4
Other administrative and managerial workers	699	53.9	12.8	81	11.6	10	7.9
Professional and engineering workers							
Researchers	487	50.9	10.1	16	3.3	4	2.7
Manufacturing engineers	2354	50.5	9.7	507	21.5	12	4.4
Architects, civil engineers, and surveyors	1652	55.9	11.7	95	5.8	10	9.2
Data processing and communication engineers	911	49.4	9.0	78	8.6	8	6.7
Other engineers	1110	51.5	10.1	128	11.5	8	5.9
Doctors	402	68.0	17.9	274	68.2	6	5.2
Nurses	481	46.8	8.8	412	85.7	7	3.1
Medical technicians	722	47.7	9.0	186	25.8	4	3.3
Social welfare specialist professionals	538	46.7	8.5	248	46.1	6	3.5
Teachers	1246	59.2	14.4	34	2.7	11	8.5
Other specialist professionals	2509	51.1	12.5	322	12.8	8	6.5
Clerical workers							
General clerical workers	1945	47.0	8.6	122	6.3	6	6.4
Sales clerks	674	53.8	11.4	32	4.7	6	7.0
Other clerical workers	1246	49.0	9.8	144	11.6	8	6.0
Sales workers							
Merchandise sales workers	1416	54.7	12.4	137	9.7	13	8.0
Sales workers	2721	57.1	11.6	89	3.3	10	8.8
Other sales workers	270	53.6	12.9	25	9.3	10	9.4
Service workers							
Care service workers	956	45.8	9.5	626	65.5	6	3.6
Food and drink preparatory workers	1007	62.4	15.4	118	11.7	15	9.0
Customer service workers	1026	53.4	12.9	357	34.8	14	6.7
Other service workers	1342	55.4	13.1	288	21.5	10	6.5
Security workers							
Self-defense officials	454	47.5	13.8	241	53.1	4	3.1
Judicial police staff, such as police officers	628	59.7	16.4	541	86.1	7	4.3
Other public security workers, such as firefighters	641	57.2	18.5	503	78.5	10	2.7
Agricultural, forestry, and fishery workers							
Agriculture workers	403	56.3	14.4	12	3.0	12	10.4
Forestry workers and fishery workers	261	51.5	14.6	24	9.2	17	8.1
Manufacturing process workers							
Product manufacturing and processing workers	2983	48.6	9.3	1441	48.3	12	4.1
Machine maintenance and repair workers	732	51.1	11.3	128	17.5	12	5.9
Other manufacturing process workers	1623	48.5	9.7	690	42.5	12	4.2
Transport and machine operation workers							
Motor vehicle drivers	1111	63.5	17.2	246	22.1	14	7.4
Other transport workers	536	50.9	13.7	292	54.5	12	5.8
Construction and mining workers							
Construction workers	778	54.3	11.4	36	4.6	10	9.3
Electric construction workers	601	54.8	13.2	96	16.0	8	8.2
Civil engineering workers	569	51.9	9.5	49	8.6	12	9.1
Other construction and mining workers	745	54.1	11.0	57	7.7	9	9.1
Carrying, cleaning packaging, and related workers							
Carrying workers, cleaning workers, and packaging workers	550	53.3	14.1	95	17.3	13	7.7

*SD* standard deviation

### Workhours/shift work and dietary behaviors

Table 3 shows the associations of workhours/shift work with dietary behaviors. After adjusting for age, income, education, occupation, partners’ age, partners’ education, partners’ occupation, and shift work, men who worked more than 65 h/week showed significantly higher odds ratios (ORs) for the various dietary behaviors than men who worked 40 h/week or less [skipping breakfast, OR 1.48 (95% confidence interval [CI] 1.38–1.60); eating out, OR 1.31 (95% CI 1.22–1.42); eating instant food, OR 1.38 (95% CI 1.28–1.48); overeating, OR 1.27 (95% CI 1.18–1.38); and eating fast, OR 1.10 (95% CI 1.02–1.19)]. The P trends for the odds of dietary behaviors according to the workhours were significant across the five indicators of dietary behaviors. With increasing workhours, the likelihood of skipping breakfast, eating out, eating instant food, overeating (P for trend < 0.001), and eating fast (P for trend = 0.015) increased, after adjusting for potential confounding factors. Regarding eating fast, only those who worked more than 65 workhours per week showed a significantly higher OR after adjusting for potential confounding factors.

**Table 3:** Associations between workhours/shift work and dietary behaviors (*N* = 39,315)

Workhours		Skipping breakfast						Eating out						Eating instant food						Overeating						Eating fast					
	*n*	Unadjusted			Adjusted^a^			Unadjusted			Adjusted^a^			Unadjusted			Adjusted^a^			Unadjusted			Adjusted^a^			Unadjusted			Adjusted^a^		
		OR	95 CI		OR	95 CI		OR	95 CI		OR	95 CI		OR	95 CI		OR	95 CI		OR	95 CI		OR	95 CI		OR	95 CI		OR	95 CI	
> 0, ≦ 40 h/week	8275	1.00			1.00			1.00			1.00			1.00			1.00			1.00			1.00			1.00			1.00		
> 40, ≦ 45 h/week	5337	0.97	0.91	1.04	1.03	0.96	1.11	1.30	1.21	1.39	1.19	1.11	1.28	1.03	0.97	1.11	1.12	1.05	1.21	1.04	0.97	1.12	1.04	0.96	1.12	0.94	0.87	1.01	0.94	0.87	1.01
> 45, ≦ 50 h/week	9513	1.08	1.02	1.15	1.11	1.04	1.18	1.19	1.12	1.27	1.15	1.09	1.23	1.09	1.03	1.16	1.10	1.03	1.17	1.09	1.02	1.16	1.11	1.04	1.19	1.02	0.96	1.09	1.02	0.96	1.09
> 50, ≦ 55 h/week	3783	1.18	1.09	1.28	1.20	1.10	1.30	1.25	1.15	1.35	1.18	1.09	1.28	1.09	1.01	1.18	1.12	1.03	1.21	1.15	1.06	1.25	1.17	1.08	1.28	1.02	0.94	1.10	0.99	0.91	1.08
> 55, ≦ 65 h/week	6441	1.39	1.30	1.48	1.36	1.27	1.46	1.39	1.30	1.49	1.26	1.17	1.35	1.15	1.07	1.22	1.17	1.09	1.25	1.12	1.04	1.20	1.14	1.06	1.23	1.07	1.00	1.15	1.03	0.95	1.10
> 65 h/week	5966	1.58	1.48	1.69	1.48	1.38	1.60	1.50	1.40	1.61	1.31	1.22	1.42	1.38	1.29	1.48	1.38	1.28	1.48	1.24	1.15	1.33	1.27	1.18	1.38	1.20	1.11	1.29	1.10	1.02	1.19
P trend			< 0.001			< 0.001			< 0.001			< 0.001			< 0.001			< 0.001			< 0.001			< 0.001			< 0.001			0.015	
Irregular shift																															
0 times/month	30,448	1.00			1.00			1.00			1.00			1.00			1.00			1.00			1.00			1.00			1.00		
> 0, ≦ 8 times/month	3822	1.24	1.16	1.32	1.33	1.23	1.44	0.97	0.90	1.03	1.04	0.96	1.13	1.35	1.27	1.45	1.37	1.27	1.48	1.07	0.99	1.15	1.03	0.95	1.13	1.07	1.00	1.16	1.01	0.93	1.10
> 8, ≦ 31 times/month	5045	1.74	1.64	1.85	1.79	1.67	1.92	0.88	0.83	0.93	1.16	1.09	1.25	1.78	1.68	1.90	1.69	1.58	1.82	0.97	0.91	1.03	0.93	0.86	1.00	0.93	0.87	0.99	0.96	0.90	1.03
P trend			< 0.001			< 0.001			< 0.001			< 0.001			< 0.001			< 0.001			0.110			0.078			0.005			0.497	

^a^Adjusted for age, income, education, occupation, partners’ age, partners’ education, partners’ occupation, and shift work or workhours

After adjusting for age, income, education, occupation, partners’ age, partners’ education, partners’ occupation, and workhours, men who had shift work more than 8 times per month showed significantly higher ORs for various dietary behaviors than those who did not have shift work [skipping breakfast, OR 1.79 (95% CI1.67–1.92); eating out, OR1.16 (95% CI 1.09–1.25); eating instant food, OR 1.69 (95% CI 1.58–1.82)]. As the frequency of shift work increased, the likelihood of skipping breakfast, eating out, and eating instant food increased (P for trend < 0.001), after adjustment for potential confounding factors. There was no significant OR for overeating and eating fast after adjustment for potential confounding factors.

### Occupations characterized by workhours/shift work and related dietary behaviors

Table 4 shows the association between dietary behaviors and the occupations characterized by long workhours and/or shift work. In some cases, although there were significant associations in the models adjusted for age, income, education, partners’ age, partners’ education, and partner’s occupation (hereafter called model 2), the statistical significance disappeared in the models adjusted for workhours and shift work in addition to other potential confounders (hereafter called model 3). Doctors, nurses, care service workers, customer service workers, and forestry and fishery workers showed higher odds of eating instant food, while the significant associations disappeared in model 3. Similarly, judicial police staff showed significantly higher odds of skipping breakfast, eating instant food, and overeating in model 2; this was not observed in model 3. Merchandise sales workers showed significantly higher odds of overeating; this was not observed in model 3. The significantly higher odds of skipping breakfast among doctors as seen in model 2 also disappeared in model 3. Some cases showed significant associations in model 3 rather than in model 2. In model 3, other public security workers, such as firefighters, showed lower odds of skipping breakfast and eating out; this was not observed in model 2.

**Table 4:** Dietary behaviors according to occupations characterized by workhours and/or shift work

	*n*	Skipping breakfast									Eating outside									Eating instant food								
		Model 1			Model 2^a^			Model 3^b^			Model 1			Model 2^a^			Model 3^b^			Model 1			Model 2^a^			Model 3^b^		
		OR	95 CI		OR	95 CI		OR	95 CI		OR	95 CI		OR	95 CI		OR		95 CI	OR	95 CI		OR		95 CI	OR		95 CI
Administrative and managerial																												
Management government officials	590	1.00			1.00			1.00			1.00			1.00			1.00			1.00			1.00			1.00		
Professional and engineering																												
Doctors (workhours, shift work)	402	1.57	1.21	2.05	1.98	1.49	2.62	1.32	0.99	1.77	3.08	2.25	4.22	1.93	1.39	2.68	1.66	1.19	2.32	1.10	0.85	1.42	1.43	1.09	1.89	1.00	0.76	1.32
Nurses (shift work)	481	3.20	2.49	4.12	2.47	1.91	3.20	1.92	1.48	2.51	0.75	0.59	0.96	0.82	0.64	1.05	0.78	0.60	1.00	1.94	1.52	2.47	1.59	1.24	2.03	1.20	0.93	1.55
Teachers (workhours)	1246	0.90	0.72	1.11	1.01	0.81	1.25	0.89	0.72	1.11	0.84	0.69	1.03	0.70	0.57	0.86	0.65	0.53	0.80	0.61	0.50	0.75	0.71	0.57	0.87	0.66	0.53	0.81
Sales workers																												
Merchandise sales workers (shift work)	1416	3.27	2.66	4.01	2.54	2.06	3.13	2.36	1.91	2.91	0.95	0.78	1.16	1.20	0.98	1.47	1.13	0.92	1.38	1.84	1.51	2.24	1.54	1.27	1.88	1.47	1.21	1.80
Service workers																												
Care service workers (shift work)	956	2.95	2.37	3.66	2.10	1.69	2.62	1.84	1.47	2.30	0.51	0.41	0.63	0.66	0.54	0.82	0.65	0.52	0.81	1.69	1.38	2.09	1.36	1.10	1.67	1.15	0.93	1.42
Food and drink preparatory (workhours, shift work)	1007	5.30	4.25	6.61	3.80	3.03	4.76	3.21	2.56	4.04	0.72	0.59	0.89	1.02	0.82	1.26	0.92	0.74	1.14	1.03	0.84	1.27	0.80	0.65	0.99	0.71	0.57	0.88
Customer service workers (shift work)	1026	5.37	4.31	6.69	3.56	2.84	4.46	2.95	2.35	3.70	0.88	0.72	1.09	1.20	0.97	1.49	1.10	0.89	1.37	1.89	1.54	2.32	1.45	1.17	1.78	1.23	1.00	1.52
Security workers																												
Judicial police staff (workhours, shift work)	628	2.11	1.67	2.66	1.87	1.48	2.38	1.23	0.96	1.57	1.64	1.29	2.09	1.65	1.29	2.11	1.43	1.11	1.84	1.78	1.42	2.24	1.69	1.34	2.13	1.16	0.91	1.46
Other public security workers (shift work)	641	1.37	1.08	1.74	1.05	0.83	1.34	0.65	0.51	0.83	0.76	0.60	0.95	0.83	0.66	1.05	0.71	0.56	0.90	2.29	1.82	2.87	1.94	1.54	2.45	1.29	1.02	1.63
Agricultural, forestry, and fishery																												
Forestry and fishery (shift work)	261	2.14	1.59	2.89	1.28	0.95	1.75	1.23	0.90	1.68	0.36	0.26	0.48	0.50	0.37	0.68	0.48	0.36	0.66	1.88	1.40	2.52	1.36	1.01	1.83	1.34	0.99	1.80
Transport and machine operation workers																												
Motor vehicle drivers (workhours, shift work)	1111	3.86	3.12	4.78	2.80	2.25	3.49	2.27	1.81	2.83	0.76	0.62	0.94	1.06	0.86	1.31	0.95	0.77	1.17	2.38	1.94	2.92	1.84	1.49	2.26	1.58	1.28	1.95
	*n*		Overeating													Eating fast												
			Model 1				Model 2^a^				Model 3^b^					Model 1					Model 2^a^				Model 3^b^			
			OR	95 CI			OR		95 CI		OR		95 CI			OR	95 CI				OR	95 CI			OR	95 CI		
Administrative and managerial																												
Management government officials	590		1.00				1.00				1.00					1.00					1.00				1.00			
Professional and engineering																												
Doctors (workhours, shift work)	402		0.86	0.6		1.12	0.99		0.75	1.32	0.88		0.66	1.18		1.17	0.89		1.55		1.22	0.91	1.63		1.15	0.85		1.55
Nurses (shift work)	481		1.35	1.03		1.77	1.22		0.93	1.60	1.23		0.93	1.63		1.18	0.90		1.53		1.10	0.84	1.43		1.11	0.85		1.46
Teachers (workhours)	1246		1.06	0.86		1.31	1.12		0.91	1.39	1.04		0.84	1.29		1.23	0.99		1.52		1.29	1.04	1.60		1.25	1.01		1.55
Sales workers																												
Merchandise sales workers (shift work)	1416		1.27	1.03		1.56	1.24		1.01	1.53	1.19		0.96	1.47		1.08	0.88		1.33		1.05	0.85	1.29		1.03	0.84		1.27
Service workers																												
Care service workers (shift work)	956		1.44	1.15		1.81	1.38		1.09	1.73	1.38		1.09	1.75		0.95	0.77		1.19		0.91	0.73	1.14		0.92	0.73		1.15
Food and drink preparatory (workhours, shift work)	1007		1.24	0.99		1.55	1.24		0.99	1.55	1.13		0.90	1.42		1.45	1.16		1.82		1.40	1.11	1.76		1.35	1.07		1.69
Customer service workers (shift work)	1026		1.12	0.90		1.39	1.10		0.88	1.37	1.07		0.85	1.34		1.11	0.89		1.39		1.07	0.85	1.33		1.06	0.85		1.33
Security workers																												
Judicial police staff (workhours, shift work)	628		1.38	1.07		1.77	1.33		1.03	1.71	1.25		0.96	1.61		1.20	0.94		1.54		1.17	0.91	1.49		1.14	0.89		1.47
Other public security workers (shift work)	641		1.07	0.84		1.36	1.00		0.78	1.27	0.98		0.76	1.26		0.97	0.76		1.23		0.92	0.72	1.17		0.91	0.72		1.17
Agricultural, forestry, and fishery workers																												
Forestry and fishery (shift work)	261		1.13	0.82		1.55	1.11		0.80	1.52	1.07		0.77	1.47		0.88	0.65		1.20		0.85	0.62	1.16		0.84	0.61		1.14
Transport and machine operation workers																												
Motor vehicle drivers (workhours, shift work)	1111		1.38	1.11		1.72	1.39		1.11	1.74	1.28		1.02	1.60		0.96	0.78		1.19		0.92	0.74	1.14		0.89	0.71		1.10

*Administrative and managerial* administrative and managerial workers; *Professional and engineering* professional and engineering workers; *Food and drink preparatory* food and drink preparatory workers; *Judicial police staff* judicial police staff, such as police officers; *Other public security workers* other public security workers, such as firefighters; *Agricultural, forestry, and fishery* agricultural, forestry, and fishery workers; *Forestry and fishery* forestry workers and fishery workers

^a^Adjusted for age, income, education, partners’ age, partners’ education, and partners’ occupation

^b^Adjusted for age, income, education, partners’ age, partners’ education, partners’ occupation, workhours, and shift work

### Occupation and dietary behaviors

Several occupations showed specific dietary behaviors even after adjusting for time-related work factors (model 3). For example, compared with management government officials, teachers were less likely to eat outside [OR 0.65 (95% CI, 0.53–0.80)] and eat instant food [OR 0.66 (95% CI, 0.53–0.81)], while they were more likely to eat quickly [OR 1.25 (95% CI, 1.01–1.55)]. Care service workers showed a greater likelihood of skipping breakfast [OR 1.84 (95% CI, 1.47–2.30)] and overeating [OR 1.38 (95% CI, 1.09–1.75)], while they were less likely to eat outside [OR 0.65 (95% CI, 0.52–0.81)]. Food and drink preparatory workers were more likely to skip breakfast [OR 3.21 (95% CI, 2.56–4.04)] and eat fast [OR 1.35 (95% CI, 1.07–1.69)], while they were less likely to eat instant food [OR 0.71 (95% CI, 0.57–0.88)]. Other public security workers, such as firefighters, were less likely to skip breakfast [OR 0.65 (95% CI, 0.51–0.83)] and eat outside [OR 0.71 (95% CI, 0.56–0.90)] and more likely to eat instant food [OR 1.29 (95% CI, 1.02–1.63)]. Motor vehicle drivers tended to skip breakfast [OR 2.27 (95% CI, 1.81–2.83)], eat instant food [OR 1.58 (95% CI, 1.28–1.95)], and overeat [OR 1.28 (95% CI, 1.02–1.60)].

## Discussion

The present study revealed that workhours and shift work were independently associated with dietary behaviors. Long workhours were positively associated with poor dietary behaviors in various aspects of eating: skipping breakfast, eating out, eating instant food, overeating, and eating fast. The frequency of shift work was also associated with poor dietary behaviors in some aspects of eating: skipping breakfast, eating out, and eating instant food more frequently. Some occupations characterized by long workhours and/or shift work tended to have specific dietary behaviors. After adjusting for workhours and shift work, in addition to other potential confounding factors, some associations remained significant while others became insignificant.

Time-related restrictions may result in specific dietary behaviors, such as missing meals (e.g., skipping breakfast), taking lesser time to eat (overeating, eating fast), and preparing meals in shorter durations (eating out, eating instant food). Very few studies have examined the association between workhours and dietary behaviors, such as eating breakfast and eating between meals [18]. Data on other dietary indicators according to workhours are sparse. This study presented clear evidence on the associations between workhours and dietary behaviors, using detailed classifications of workhours and various dietary indicators, with a large number of study participants. With increasing workhours, the trends of skipping breakfast, eating out, and eating instant food seemed to increase. Overeating and eating fast were also positively associated with workhours to some extent. These results suggest that long workhours lead to poor dietary behaviors, through missed meals, shorter times taken to eat, and shorter times taken to prepare meals.

However, regarding shift work, the same trends were not always observed. The frequency of shift work was not positively associated with overeating and eating fast but was positively associated with skipping breakfast, eating out, and eating instant food. Shift workers have been well-known to have irregular eating patterns [6], and our study’s findings confirm this. A novel finding of our study is that we found no positive association between the frequency of shift work and the manner of eating, namely, taking a shorter time to eat. Shift workers may consume meals in a calm manner, provided they have enough time to take a break. Given this evidence, it is possible that having long workhours, rather than shift work, was more strongly linked to poor dietary behaviors in our study.

This study also evaluated trends of dietary behaviors across occupations characterized by long workhours and shift work. In some cases, the observed significant associations between occupations and dietary behaviors disappeared after adjusting for workhours and shift work in addition to other potential confounding factors, suggesting that long workhours/shift work could affect dietary behaviors. Doctors, nurses, care service workers, customer service workers, judicial police staff, and forestry and fishery workers, for example, may be less likely to eat instant food provided they have enough time for meals. In other cases, the associations remained significant after adjusting for workhours and shift work in addition to other potential confounding factors. These findings may be explained through factors influenced by the occupations, such as food environment. For example, teachers, food and drink preparatory workers, and other public security workers such as firefighters showed good dietary behaviors in certain aspects, although they also reported one or more poor dietary behaviors. One possibility is that workplace food environments are well-developed in such occupational groups.

In workplaces such as schools, teachers may utilize the lunch system or cook their own meals. Accordingly, teachers showed a lower likelihood of eating instant food and eating outside. Our previous study reported higher intakes of dairy products and calcium among teachers [19], also lending support to the contribution of school lunch to dietary behaviors. In contrast, a poor food environment in the workplace, as well as work situation, may contribute to poor dietary behaviors; for example, motor vehicle drivers demonstrated a higher likelihood of skipping breakfast, eating instant food, and overeating, which may be due to poor food availability and accessibility along their driving route and traffic situations.

Overall, shorter workhours and/or reduced shift frequency may improve dietary behaviors. Improving other factors influenced by occupation, including food environment in the workplace, may also lead to favorable dietary behaviors; providing enough time and space and serving healthy food and drink in the workplace are important. If a reduction in shift work is not foreseen, at least an effort to improve the food environment through healthier food and drink options in workplace cafeterias and vending machines can be beneficial for the workers’ health. In previous studies, the presence of cafeterias [20, 21] and vending machines [20] was associated with workers’ diets. Workers can learn healthy diet choices through the use of such food facilities if they provide healthy food and drinks. Monitoring time-related factors and dietary behaviors at each workplace, as well as developing food environment and nutrition education [22] for workers as a public health action, would also be beneficial to promote workers’ health.

### Limitation

This study has several limitations that should be acknowledged. First, the study sample comprised only expectant fathers. Their dietary behaviors may be affected by their partners’ pregnancy, encouraging them to change their health-related behaviors. In addition, our participants may be more interested in health and have higher levels of knowledge on the same. Therefore, it may be difficult to generalize the results of this study directly. Second, the analysis was performed using data from self-reported questionnaires, suggesting that over- or underreporting should be considered in interpreting the results. In addition to time spent eating, sleeping time and resting times may be influenced by time-related factors and affect health problems; information on such lifestyle factors should also be assessed by the questionnaires. Third, our results showed simple associations between working conditions and dietary behaviors due to the cross-sectional study design. Detailed data on the causal mechanisms of the findings remain unclear; further longitudinal studies may be helpful in clarifying them. Additionally, the present study did not assess food quality and meal timing, energy balance, and nutrient intakes, which should be considered according to work schedule. Fourth, the questionnaire did not distinguish between paid and unpaid work among self-employed individuals, which might have an impact on dietary behaviors. Finally, future studies should evaluate women’s diets according to occupation given the increasing number of employed women in Japan [23]. In light of women’s social progress, the impact of time-related work factors would be greater.

## Conclusion

In summary, this study examined the associations between time-related work factors and dietary behaviors among male workers in Japan. Both longer workhours and having shift work were associated with more frequently skipping breakfast, eating out, and eating instant food after adjusting for potential confounders. The likelihoods of overeating and eating fast were also higher among those who worked long hours. Several occupations involving long workhours and/or shift works showed specific dietary behaviors, some of which were changed after the adjustment of workhours and shift work, in addition to other potential confounding factors. Time-related work factors, as well as other factors influenced by occupation, may have a variable impact on workers’ dietary behaviors.

## Abbreviations

JECS: Japan Environment and Children’s Study
